# Computer-Guided Development of Hyperbranched Modified Starch-Based Adhesives

**DOI:** 10.3390/polym17131812

**Published:** 2025-06-29

**Authors:** Hongjian Yu, Jiang Chang, Wenrui Chi, Shuzhen Gao, Jie Liu, Yin Tang

**Affiliations:** College of Light Industry and Textile, Qiqihar University, Qiqihar 161006, China; chji0824@163.com (J.C.); 03810@qqhru.edu.cn (W.C.); gaoshuzhen@126.com (S.G.); liujie0452@126.com (J.L.); tangyin0001@163.com (Y.T.)

**Keywords:** starch, hyperbranching, simulation, performance

## Abstract

In this study, a novel starch-based adhesive (SBA) was proposed, which mainly involved the synthesis of a carboxyl-terminated hyperbranched polymer using bisphenol A diglycidyl ether (DGEBA) and citric acid as raw materials. Subsequently, starch was modified through hyperbranching to enhance the shear strength and water resistance of the SBA. For this purpose, the feasibility of the reaction between DGEBA and citric acid was analyzed using quantum mechanical simulations. Subsequently, both substances were simulated to synthesize carboxyl-terminated hyperbranched polymers with different ratios. Starch was modified through hyperbranching to establish various models of SBAs, and their properties were estimated using molecular dynamics simulations. Theoretical analysis indicates that a DGEBA-to-citric acid ratio of 3:7 yields a SBA with relatively optimal properties. The solubility parameter of this adhesive is 19.05 (J/cm^3^)^1^/^2^, suggesting strong intermolecular interactions between the hyperbranched polymer and starch. The synthesized adhesive exhibits high cohesive strength, with an estimated water contact angle of up to 138°, indicating good hydrophobicity. Furthermore, the system demonstrates favorable mechanical performance, with a shear modulus of 4.34 GPa and a bulk modulus of 8.80 GPa. Additionally, at this ratio, the SBA exhibits a relatively high interaction energy of −408.01 kcal/mol with the cellulose substrate, suggesting that the adhesive possesses favorable shear strength.

## 1. Introduction

In recent years, there has been a global push for green development, with “wood replacing plastic” and “wood replacing steel” being seen as important directions for future environmentally friendly and low-carbon development [1,2,3]. Adhesives are essential in the process of preparing wood products, as they can be used to transform wood chips and sawdust into particleboard and plywood, which have a wide range of applications in construction, home furnishings, and other areas. Currently, formaldehyde-based resin adhesives dominate the market for wood adhesives, accounting for over 80% of the market share [4]. The continuous use of such adhesives not only exacerbates the depletion of petroleum resources but also releases volatile substances, such as formaldehyde, which cause serious harm to both the ecological environment and people’s physical health [5,6]. Therefore, the utilization of biomass materials for the preparation of eco-friendly adhesives as a substitute for formaldehyde-based adhesives has garnered widespread attention.

Starch, a type of polysaccharide polymer, has a large number of hydroxyl groups on its surface and exhibits excellent hydrogen bonding with the cellulose matrix. As a result, starch is extensively applied in the field of wood processing [7,8,9,10]. However, the multi-hydroxyl structure of starch provides it with a certain shear strength but also leads to issues such as poor water resistance [5,11]. Furthermore, the bonding force between starch and substrates relies solely on hydrogen bonding, which fails to meet people’s demands. To improve the performance of SBA, crosslinking modification is an effective approach. For example, epoxy compounds [12,13], furfural [14], and isocyanates [15,16,17] can serve as crosslinking agents for hydroxyl groups. This not only increases the degree of crosslinking but also reduces the number of hydrophilic groups, thereby enhancing the shear strength and water resistance of SBA [18,19,20]. The hyperbranched polymers are highly branched polymer structures that mainly expand outward from a reactive molecule at the center [21]. The interior of hyperbranched molecules is mostly hollow with multiple branching points, containing numerous reactive end groups that exhibit high activity. This characteristic has attracted significant attention in fields such as coatings and adhesives. Yi Zhang et al. [22] synthesized a terminal amino-functionalized hyperbranched structure using succinic anhydride and diethylenetriamine as raw materials, which reacted with oxidized starch to form hyperbranched amino–starch. Finally, this amino–starch was mixed with soy protein to prepare a soy protein-based adhesive. This adhesive exhibits a highly crosslinked structure and excellent toughness, and more importantly, it possesses outstanding shear strength and water resistance (1 MPa). Tao Jin et al. [23] prepared hyperbranched polyols by conducting a nucleophilic addition reaction between succinic anhydride and diethanolamine, followed by esterification with citric acid to obtain a citric acid-based hyperbranched polyester resin. The resin exhibited excellent bonding performance, particularly in terms of water resistance, as the wet shear strength (in hot water and boiling water) of the produced plywood samples exceeded 0.7 MPa. Additionally, the authors of [24] utilized hyperbranched polyamide to combine with dialdehyde starch through a Schiff base reaction and introduced glucose for secondary crosslinking, resulting in a SBA with a dual crosslinked structure. Even after immersion in boiling water, the wet strength of the produced plywood remained at 1.26 MPa.

Computer simulation is an interdisciplinary technique based on mathematical, physical, and chemical theories that allows for the exploration of chemical reactions at a microscopic level. Molecular dynamics simulation focuses specifically on studying geometric configurations and reaction mechanisms at the molecular level. Additionally, molecular simulation enables researchers to optimize systems and narrow down the experimental scope, thereby improving work efficiency. This provides significant advantages for scientific research. Manjinder Singh et al. [25] proposed the use of epoxidized linseed oil as a substitute for 2-ethylhexyl acrylate in pressure-sensitive adhesives to develop environmentally friendly and sustainable adhesive materials. Molecular dynamics simulations were employed to evaluate intrinsic properties such as system density, solubility parameters, and glass transition temperature, which were consistent with previous literature reports. Furthermore, molecular dynamics revealed that epoxidized linseed oil can provide higher binding energy for pressure-sensitive adhesives, significantly increasing both shear modulus and bulk modulus. This indicates the high potential of this adhesive material in green design applications within the food packaging and healthcare industries. In their study on SBA, Chen et al. [26] employed computer simulations to investigate the self-assembly behavior of vinyl acetate grafting onto starch molecules. The results indicated that the primary grafting site was the C2 position of glucose units. Additionally, compared to native starch, the grafted starch exhibited fewer hydrogen bonding interactions, lower orderliness, and higher extensibility, which were consistent with experimental findings.

Based on the aforementioned background, this study employs bisphenol A diglycidyl ether (DGEBA) and citric acid as raw materials to synthesize carboxyl-terminated hyperbranched polymers via a ring-opening addition reaction. Subsequently, starch modification is achieved through esterification reactions utilizing the synthesized hyperbranched polymers. This modification aims to impart enhanced shear strength and water resistance to SBAs. This is the first report on the application of this specific hyperbranched system for starch modification. The reactivity of carboxyl-terminated hyperbranched polymers was enhanced by synthesizing them using epoxy resin (DGEBA) and citric acid as raw materials in this study. Subsequently, the modified starch was hyperbranched to improve the shear strength and water resistance of the SBA, which represents the first application of this hyperbranched system for starch modification. Quantum mechanical simulations were conducted in Materials Studio (MS) to explore the electrostatic potential and frontier molecular orbitals of DGEBA and citric acid, aiming to assess the feasibility of hyperbranched polymerization for these two monomers. Simulation analysis was then conducted on the developed SBA system to assess properties such as density (ρ), solubility parameter (δ), water contact angle, and interaction energy under different formulations, with the aim of evaluating its adhesion performance and water resistance. The theoretical insights gained are intended to guide subsequent experimental work.

## 2. Results and Discussion

### 2.1. Electrostatic Potential Diagram and Charge Distribution of DGEMA and Citric Acid

**Figure 1:**
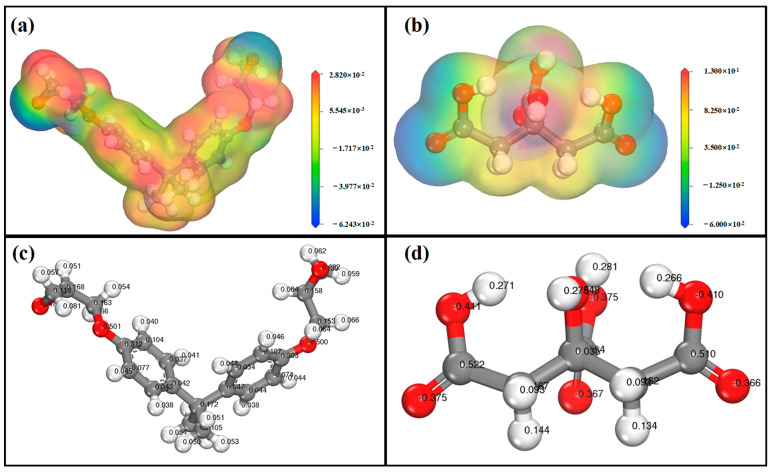
(**a**) Electrostatic potential diagram of DGEMA; (**b**) electrostatic potential diagram of citric acid; (**c**) charge value of DGEMA; (**d**) charge value of citric acid.

According to reports, the electrostatic potential can reflect the reactivity of material structures to some extent. The electrostatic potential map visualizes the electron density of material structures, providing a better understanding of microconcepts for molecules and polymers [27,28]. As depicted in Figure 1, the electrostatic potential map delineates the charge distribution of the substance, as well as the regions for nucleophilic and electrophilic reactions, which are related to molecular conformation and size [29,30]. In the electrostatic potential diagram, red represents the low-potential region for nucleophilic reactions, while blue represents the high-potential region for electrophilic reactions. By conducting computer simulations, visualized electrostatic potential values of citric acid and DGEBA have been obtained. Due to the higher electronegativity of oxygen atoms, electrons in the epoxy groups are concentrated on the oxygen atom in DGEBA, resulting in lower electron density on carbon atoms. Combined with the molecular charge of DGEBA, the oxygen atoms have charges of −0.478 and −0.482, respectively, while the carbon atoms in the epoxy group have charge values ranging from 0.118 to 0.168, providing favorable nucleophilic reaction sites. Similarly, in the electrostatic potential diagram of citric acid, the blue area is mainly contributed to by oxygen atoms in the molecule, particularly those in the hydroxyl group with charges of −0.411, −0.410 and −0.375, respectively, which can act as electron donors. These findings indicate that both DGEBA and citric acid are prone to chemical reactions.

### 2.2. Discussion on the Frontier Molecular Orbitals of DGEMA and Citric Acid

**Figure 2:**
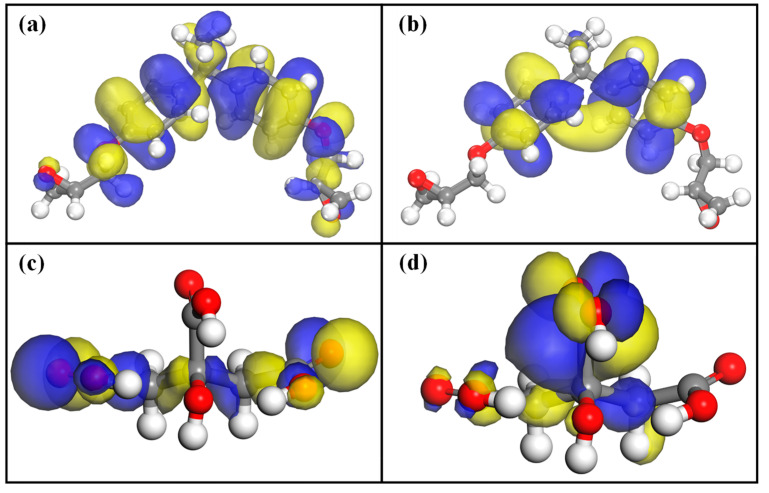
Frontier molecular orbital simulation results: (**a**) HOMO orbit of DGEBA; (**b**) LUMO orbit of DGEBA; (**c**) HOMO orbit of citric acid; (**d**) LUMO orbit of citric acid.

Based on density functional theory, the geometric configurations of citric acid and DGEBA molecular models were optimized. The numerical values of HOMO, LUMO, and Fukui indices were calculated through frontier molecular orbital analysis and Fukui function, as presented in Table 1 and Figure 2. The reactivity of molecules can be determined by the frontier molecular orbitals, with smaller energy gaps indicating higher reactivity and lower stability [31]. The HOMO orbital is the highest occupied molecular orbital, which can function as an electron donor, while the LUMO orbital is the lowest unoccupied molecular orbital, which can function as an electron acceptor, thus revealing reaction mechanisms.

From Figure 2, it can be observed that the HOMO and LUMO orbitals of DGEBA are primarily distributed around the benzene ring, indicating that this region can function as both electron trap sites and hole trap sites. This observation is consistent with the findings of Yushun Zhao’s research [32]. However, the 2p orbitals of carbon atoms in the benzene ring and the 2p orbitals of lone pair electrons in oxygen ether atoms form a conjugated system in the bonding orbitals of DGEBA molecules, which increases electron delocalization and lowers the energy of the molecular system. As a result, this part becomes relatively stable and less reactive. However, the bond angle within the epoxide ring is approximately 60° [33], which creates significant tension and has the potential to cause ring opening. Considering the molecular charge of DGEBA, it can be predicted that the carbon atoms in this region are prone to nucleophilic reactions. In the visualized molecular orbitals, the two carboxyl groups of citric acid have HOMO orbitals (Figure 2c), which can donate electrons, indicating that electrophilic reactions are likely to occur at these sites. On the other hand, although there are LUMO orbitals in another carboxyl group of citric acid, the oxygen atom in this carboxyl group has a higher electronegativity and a larger electron cloud density, making it also susceptible to chemical reactions with epoxy groups in DGEBA.

Furthermore, the intermolecular energy gaps of the molecules also serve as an indicator of their reactivity. According to Table 2, the intermolecular energy gaps between DGEBA and citric acid are 0.142973 and 0.169256, respectively, suggesting that both molecules exhibit strong reactivity. The chemical potential (μ) can express electron exchange in molecular structures, and the more negative the value of the chemical potential, the easier it is for electron transfer to occur [34]. On the other hand, the lower hardness and higher flexibility also indicate excellent charge transfer ability. Therefore, in conclusion, citric acid can form hyperbranched polymers with DGEBA, as proven through theoretical analysis. By crosslinking the prepared hyperbranched polymer with starch, the shear strength and water resistance of starch can be improved.

### 2.3. Simulation of Molecular Density and Solubility Parameters of Starch-Based Adhesives

Molecular dynamics simulation can be used to estimate the fundamental properties of polymers, thereby enabling researchers to accurately assess polymer performance. Pratik Sanjiv Kasbe et al. [35] discovered that the computer-simulated density of adhesives closely matched experimental values, significantly enhancing research efficiency. The density and solubility parameters of five systems were calculated in this study, as shown in Table 3. The density values of the simulated SBAs varied between 1.09 and 1.25 g/cm^3^ for different material ratios. When the ratio of DGEBA to citric acid was 3:7, the adhesive had the highest density value, while its solubility parameter was 19.05 (J/cm^3^)^1/2^. This indicates that the carboxyl-terminated hyperbranched polymer synthesized from DGEBA and citric acid exhibits strong interaction with the hydroxyl groups of starch, thereby enhancing their interfacial interaction and improving the water resistance of the SBA. Although the solubility parameter of the St-DGEBA_1_-CA_9_ system is the highest, the ratio of DGEBA to citric acid in this system is only 1:9, resulting in a lower degree of branching and inadequate water resistance for starch. The higher solubility parameter of the St-DGEBA_1_-CA_9_ system may be attributed to both intramolecular and intermolecular hydrogen bonding within starch molecules.

### 2.4. Interaction Energy Analysis

The shear strength of substrates such as cellulose is an important criterion for evaluating the quality of SBA, which can be assessed through the molecular dynamics simulation of interaction energy. The interaction energy is influenced by both the chemical composition of the adhesive and the surface characteristics of the bonded substrate [36,37]. In this study, different hyperbranched systems were simulated using DGEBA and citric acid, and then crosslinked with starch to evaluate their interaction energy with the cellulose matrix. The results shown in Table 4 were obtained from 60-frame NVT ensemble dynamics. Different hyperbranched systems exhibit varying interaction energies. When the ratio of DGEBA to citric acid is 1:9, the interaction energy is low due to the limited presence of epoxy groups in this system, which makes it challenging for DGEBA and citric acid to form highly branched hyperbranched polymers. Even if subsequent esterification modification is performed on starch, it cannot generate SBA with high cohesive strength because there are fewer crosslinking points. Therefore, the interaction energy with the cellulose matrix remains low. The interaction energy can also be observed to change by altering the ratio of DGEBA to citric acid. Particularly, when the ratio is 3:7, the resulting hyperbranched system exhibits more branching points and can generate a larger number of reactive end groups. This leads to an increased number of crosslinking positions with the starch’s macromolecular skeleton and enhances cohesion. On the other hand, the increase in carboxyl group content in the hyperbranched system enhances hydrogen bonding between SBA and cellulose, thereby improving the interaction energy between the adhesive and substrate, with a maximum negative value of −408.01 kcal/mol. However, when the ratio of DGEBA to citric acid is 5:5, the interaction energy reaches its lowest point at only −199.54 kcal/mol. It is possible that, in this system, the lower carboxyl content provides fewer reactive sites for interacting with starch, resulting in a decreased crosslink density in the adhesive. The decrease in cohesion reduces the interaction between the adhesive and the cellulose matrix.

### 2.5. Water Contact Angle of Starch-Based Adhesives

**Figure 3:**
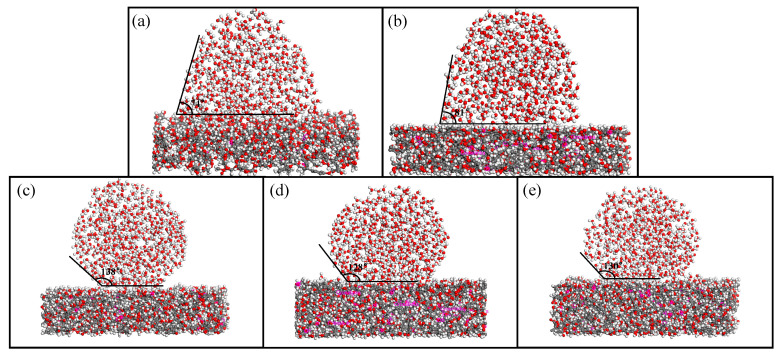
Simulation of water contact angle: (**a**) St-DGEBA_1_-CA_9_ adhesive; (**b**) St-DGEBA_2_-CA_8_ adhesive; (**c**) St-DGEBA_3_-CA_7_ adhesive; (**d**) St-DGEBA_4_-CA_6_ adhesive; (**e**) St-DGEBA_5_-CA_5_ adhesive.

To understand the effect of hyperbranched polymers with different degrees of branching on the water contact angle, the wettability of liquid water droplets on the surfaces of various SBAs was studied using molecular dynamics simulation (Figure 3).

Among them, St-DGEBA_1_-CA_9_ and St-DGEBA_2_-CA_8_ exhibit relatively low contact angles of 74° and 81°, respectively, indicating poor water resistance. This is primarily due to the presence of fewer epoxy groups in this system, resulting in a reduced algebraic degree of hyperbranched polymer formation during the ring opening reaction between DGEBA and citric acid. Consequently, the number of terminal carboxyl groups is low, leading to fewer crosslinking sites with starch and an inability to effectively reduce the number of hydrophilic hydroxyl groups. Moreover, the crosslinking density within the resulting SBA system is low, making it difficult to resist the penetration of water molecules. Therefore, starch is not endowed with satisfactory water-resistant properties. By adjusting the content of DGEBA and citric acid, hyperbranched polymers with different generations can be prepared. It can be observed that the wetting properties of water droplets on SBA also change accordingly. Among them, the water contact angles of St-DGEBA_3_-CA_7_, St-DGEBA_4_-CA_6_, and St-DGEBA_5_-CA_5_ adhesives are all greater than 90°. Due to the increase in the number of epoxy groups, the degree of branching also increases, resulting in a higher availability of crosslinking sites for starch reaction and the formation of a denser crosslinked network structure. This prevents water molecules from infiltrating [38]. In particular, when the ratio of DGEBA to citric acid is 3:7, the water contact angle reaches 138°. This may be attributed to a more suitable balance between epoxy and carboxyl groups in this system, leading to a better degree of branching and reduced hydroxyl groups from hydrophilic functional groups. Therefore, St-DGEBA_3_-CA_7_ exhibits good hydrophobicity.

### 2.6. Simulation of Mechanical Properties of Starch-Based Adhesives

**Figure 4:**
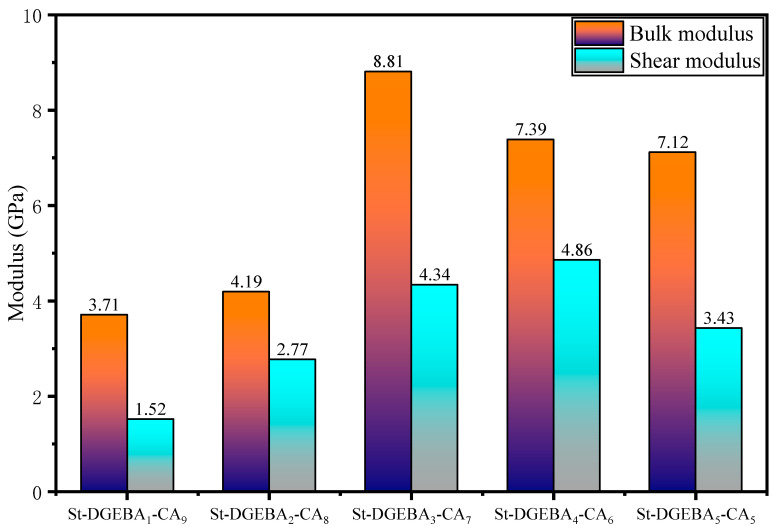
Mechanical properties of starch-based adhesives.

The shear modulus and bulk modulus of SBA were estimated and analyzed using the methodology outlined in Section 2.6, enabling the calculation and prediction of mechanical properties for various SBAs. The obtained results are presented in Figure 4. It is evident that there are significant variations in the shear modulus and bulk modulus of SBA across different systems. This suggests that hyperbranched structures of varying branching generations not only influence the water resistance of SBAs but also serve as critical determinants of their mechanical properties. The higher the bulk modulus of a SBA, the greater its ability to resist volumetric compressive deformation. This implies that an increased bulk modulus corresponds to a higher crosslinking density between molecular chains or stronger hydrogen bonding interactions, which in turn enhances the cohesive strength. The shear modulus reflects the material’s resistance to interlayer slippage. A high shear modulus indicates the restricted movement of molecular segments and a stronger cohesive structure with improved resistance to shear failure. The relatively high cohesive strength of SBA allows the adhesive to bear mechanical loads through its internal structure while simultaneously forming a stable interfacial bond with the substrate, thereby improving overall bonding performance. Combined with Section 2.5, it can be inferred that St-DGEBA_1_-CA_9_ exhibits low water resistance, volume modulus, and shear modulus due to its lower branching degree. This indicates a reduced crosslinking density between the hyperbranched system and starch, making it challenging to achieve good mechanical properties and water resistance in SBA. In the St-DGEBA_3_-CA_7_ adhesive, the hyperbranched system has abundant carboxyl end groups that undergo esterification reactions with hydroxyl groups in starch molecules. This increases crosslinking density and subsequently enhances the shear modulus and bulk modulus of SBA [39]. Furthermore, the carboxyl end groups of the hyperbranched system form numerous hydrogen bonds with hydroxyl groups in starch, resulting in intramolecular and intermolecular interactions within the system that further enhance its modulus.

Finally, by combining Section 2.3 and Section 2.4, it can be observed that when the ratio of DGEBA to citric acid is 3:7, the crosslinked simulated hyperbranched system exhibits a high degree of branching, providing numerous terminal carboxyl reaction sites. This increases its crosslinking density with starch and consequently enhances the interaction energy between SBA and the cellulose matrix [40]. Additionally, this system imparts a higher modulus and better water resistance to the adhesive.

## 3. Conclusions

The present study employs computer simulations to synthesize a carboxyl-terminated hyperbranched polymer through crosslinking DGEBA with citric acid as a reaction monomer, applying it to modify starch and establish various SBAs systems. Firstly, the feasibility of the reaction between DGEBA and citric acid was evaluated using quantum mechanical simulations. An analysis of the electrostatic potential map revealed a lower electron density in the carbon atoms of the epoxy group, with charge values primarily ranging from 0.118 to 0.168, which provides favorable nucleophilic reaction sites. The oxygen atoms in the hydroxyl groups of citric acid have charges of −0.411, −0.410, and −0.375, respectively, indicating their ability to donate electrons and suggesting that DGEBA can readily undergo a chemical reaction with citric acid. In addition, the molecular orbital theory of DGEBA and citric acid also confirms this point, with energy gaps of 0.142973 and 0.169256, respectively, proving the strong reactivity of both molecules. Furthermore, their low hardness and high flexibility reflect their excellent charge transfer ability. On the other hand, the molecular dynamics of different SBAs systems were studied. The results show that when the ratio of DGEBA to citric acid is 3:7, the degree of branching in the hyperbranched polymer improves and its interaction force with starch reaches a maximum. Under this system, the cohesive energy density of the SBA is 19.05 (J/cm^3^)^1/2^, indicating that the adhesive possesses good shear strength and water resistance. This is further supported by estimating its water contact angle and calculating the interaction energy with cellulose as the substrate. The simulation of mechanical performance also indicates that a higher degree of branching significantly enhances the shear modulus and bulk modulus of the adhesive. In general, this computer simulation-based study provides theoretical guidance for the development of new SBA and has potential in areas such as sustainable development.

## 4. Computational Studies

### 4.1. Calculation of Electrostatic Potential Between DGEBA and Citric Acid

In MS, a molecular model of DGEBA and citric acid was constructed, and GGA and BLYP hybrid functionals were used for geometry optimization in the DMol3 module. Meanwhile, a polarized double numerical quality basis set (DNP) is employed with atomic charges set to 0 to calculate the energy of different systems. Subsequently, in the analysis section of DMol3, the electrostatic potential and atomic charges of DGEBA and citric acid were analyzed. Additionally, density functional theory was employed to successfully investigate the chemical reactivity and selectivity of the substances. Therefore, in this module, calculations were also performed for the highest occupied molecular orbital (HOMO) and lowest unoccupied molecular orbital (LUMO), as well as Fukui indices for both molecules, to reveal their reactivity [41]. Through E_HOMO_ and E_LUMO_, several other quantum numbers related to electronic reactivity can also be calculated. These include the energy gap ΔE_GAP_, hardness (η), softness (σ), and chemical potential (µ).

### 4.2. Construction of Hyperbranched Starch-Based Adhesive Model

A molecular model of DGEBA and citric acid is constructed using MS software, and the “smart” approach in Compass II force field is applied for geometric optimization with 5000 iterations. To ensure rapid crosslinking between DGEBA and citric acid, unit cells are constructed in the Amorphous Cell module using optimized components of DGEBA and citric acid in different proportions, with an initial density set at 1 g/cm^3^. Then, the established periodic cell will undergo crosslinking simulation in an NVT ensemble at a temperature of 325 K, with a time step of 1 fs and a total simulation time of 20 ps. In the crosslinking process, a distance-controlled probability bonding method is employed, with the epoxy groups on DGEBA and the hydroxyl groups on citric acid set as active functional groups. When the functional groups are within a critical radius of 3.5–10 Å, there is a 50% probability of a crosslinking reaction occurring. As the program progresses, the crosslinking density of the system gradually increases. When the degree of crosslinking reaches 90%, the program is terminated [42], resulting in a hyperbranched model with carboxyl end groups.

**Figure 5:**
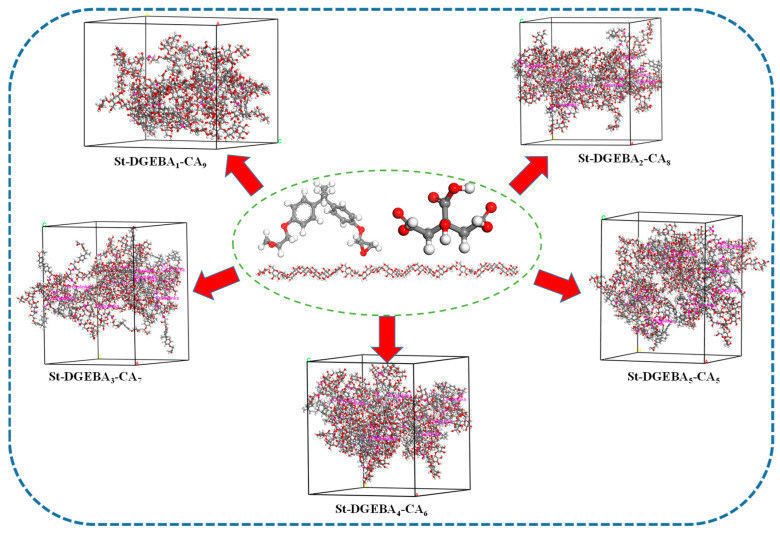
Model diagram of different starch-based adhesives.

In the MS copolymer options, construct a model with 30 glucose units to represent starch and perform geometric optimization using the method described above. Then, combine the carboxyl groups in the obtained hyperbranched model with the hydroxyl group on C6 of the starch model to create different hyperbranched SBAs, with molecular ratios as shown in Table 2. Subsequently, use the Amorphous Cell module to build periodic cells for each adhesive system (Figure 5) for further calculations.

### 4.3. Calculation of Molecular Density and Solubility Parameters

The five SBAs systems will undergo geometric optimization in the Forcite module to reduce the system’s energy. Then, each system will be annealed under NVT ensemble conditions at temperatures ranging from 300 K to 700 K, with a total of 25 cycles. The molecular dynamics simulations were then performed on each system using the Compass II force field. The NPT ensemble was selected as the primary ensemble, with a temperature of 325 K and a total simulation time of 300 ps. In addition, a Nose thermostat with a Q ratio of 0.01 was utilized for temperature control, while a Berendsen barostat with an attenuation constant of 0.1 ps was employed for pressure control. After achieving system equilibrium, the cohesive energy density of different adhesive systems can be calculated [43]. Additionally, the solubility parameter of the system can also be determined using cohesive energy density, which is used to assess compatibility between hyperbranched polymers and starch.

### 4.4. Calculation of Interaction Energy

**Figure 6:**
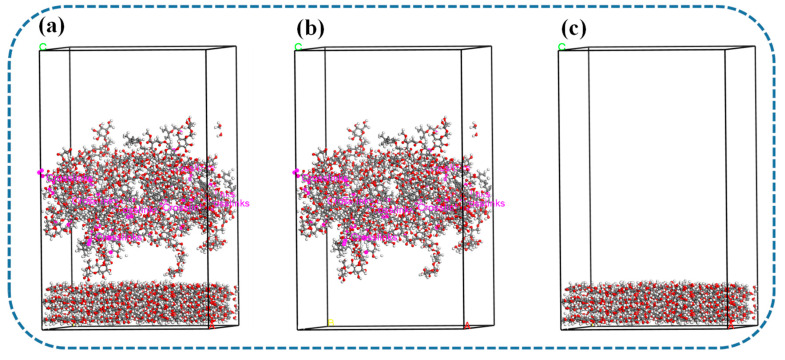
Diagram illustrating the interaction energy. (**a**) Total potential energy between starch-based adhesive and cellulose substrate; (**b**) potential energy of starch-based adhesive alone; (**c**) potential energy of cellulose substrate alone.

The interaction energy is an important parameter for evaluating the bonding performance of the developed SBA. Since this study mainly focuses on plywood preparation, cellulose is used as a substrate to assess the bonding performance between the SBA and wood, as depicted in Figure 6. To ensure the smooth progress of molecular dynamics simulations, geometric optimization was performed on both the SBA system and the cellulose substrate using the Compass II force field in the Forcite module. Then, the Build Layers tool was applied to place the adhesive and cellulose substrate in the same unit cell, creating a two-layer system with a fixed substrate structure. This allows for free interaction between surface molecules of the adhesive and cellulose substrate. Subsequently, a geometric optimization was performed on the entire system, followed by molecular dynamics simulations under the NVT ensemble at a temperature of 300 K for a total simulation time of 300 ps. The interaction energy between the SBA and cellulose substrate was calculated using the following formula [44]:

E_Interaction_ = E_Total_ − (E_SBA_ + E_Cellulose_) (1)

In Equation (1), E_Interaction_ represents the interaction energy between the SBA and cellulose substrate; E_Total_ represents the total potential energy of the simulated system; and E_SBA_ and E_Cellulose_, respectively, represent the potential energies of the independent simulation systems for adhesive and cellulose substrate.

### 4.5. Calculation of Water Contact Angle

The water resistance of SBA is a crucial factor that affects shear strength. Therefore, this study evaluated the adhesive’s water resistance by simulating the interaction between 500 water molecules and the adhesive substrate using molecular dynamics. Firstly, different SBAs were compressed and selected to modify cell states and stabilize them. Then, the water molecules were introduced into the model using Build Layers, and geometric optimization was performed on the entire unit. Subsequently, the force field was set to Compass II, and dynamic calculations were carried out under NVT ensemble with a temperature maintained at 300 K using a Nose thermostat. The total simulation time was 100 ps for calculating the water contact angle of SBA [45].

### 4.6. Mechanical Property Calculation

A superior SBA should also possess good mechanical properties, with the shear modulus (G) representing the polymer material’s ability to resist shear deformation and determining the stiffness of the adhesive. Meanwhile, the bulk modulus (K) represents the material’s ability to resist compressive deformation. After conducting molecular dynamics simulations on various systems, the mechanical properties were virtually simulated using a maximum strain amplitude of 0.003. The method of minimizing strain energy should be applied to calculate the elastic constants of the adhesive, which can be estimated by calculating the Lame constants in the stiffness matrix. The Lame constants are further divided into λ and μ values, and the G value and K value of SBA can be calculated using the following formula [43]:

$$C=\left[\begin{array}{cccccc} 2\mu +\lambda & \lambda & \lambda & 0 & 0 & 0\\ \lambda & 2\mu +\lambda & \lambda & 0 & 0 & 0\\ \lambda & \lambda & 2\mu +\lambda & 0 & 0 & 0\\ 0 & 0 & 0 & \mu & 0 & 0\\ 0 & 0 & 0 & 0 & \mu & 0\\ 0 & 0 & 0 & 0 & 0 & \mu \end{array}\right]$$

G=μ

$$K=\lambda +\frac{2}{3}\mu$$

## Figures and Tables

**Table 1:** Factors affecting the chemical reactions of DGEMA and citric acid determined based on frontier molecular orbital simulations.

Properties of Chemical Reactivity	Symbol	DGEBA	Citric Acid
Energy of HOMO (eV)		−0.186773	−0.247157
Energy of LUMO (eV)		−0.0438	−0.077901
Energy gap	ΔE_GAP_ = (E_LUMO_ − E_HOMO_)	0.142973	0.169256
Ionization energy (I)	−E_LUMO_	0.186773	0.247157
Electron affinity (A)	−E_HOMO_	0.0438	0.077901
Hardness (η)	(I − A)/2	0.0714865	0.084628
Softness (σ)	1/η	13.9887	11.81642
Chemical potential (μ)	−(I + A)/2	−0.115287	−0.162529
Electronegativity (χ)	−μ	0.1152865	0.162529

**Table 2:** Starch-based adhesives of different systems.

SBAs systems	DGEBA	Citric Acid	Starch
St-DGEBA_1_-CA_9_	1	9	10
St-DGEBA_2_-CA_8_	2	8	10
St-DGEBA_3_-CA_7_	3	7	10
St-DGEBA_4_-CA_6_	4	6	10
St-DGEBA_5_-CA_5_	5	5	10

**Table 3:** Simulated density and solubility parameters of different starch-based adhesive systems.

SBAs Systems	DGEBA	Citric Acid	Starch	ρ (g/cm^3^)	δ (J/cm^3^)^1/2^
St-DGEBA_1_-CA_9_	1	9	10	1.22	20.64
St-DGEBA_2_-CA_8_	2	8	10	1.23	16.13
St-DGEBA_3_-CA_7_	3	7	10	1.25	19.05
St-DGEBA_4_-CA_6_	4	6	10	1.09	14.53
St-DGEBA_5_-CA_5_	5	5	10	1.11	16.04

**Table 4:** Distribution of energy parameters (in kcal/mol) of the interaction between different starch-based adhesives and cellulose matrix.

System	E_Total_	E_Adhesives_	E_Base material_	E_Interaction energy_
St-DGEBA_1_-CA_9_/Base material	11,658.02	5295.53	6566.13	−203.64
St-DGEBA_2_-CA_8_/Base material	12,387.63	6149.69	6566.23	−328.29
St-DGEBA_3_-CA_7_/Base material	13,902.80	7745.18	6565.63	−408.01
St-DGEBA_4_-CA_6_/Base material	14,508.44	8234.75	6566.32	−292.63
St-DGEBA_5_-CA_5_/Base material	14,815.37	8448.20	6566.71	−199.54

## Data Availability

Data are contained within the article.
